# Thermoreversible Reverse-Phase-Shift Foam for Treatment of Noncompressible Torso Hemorrhage, a Safety Trial in a Porcine Model

**DOI:** 10.1093/milmed/usac206

**Published:** 2022-07-12

**Authors:** Ross I Donaldson, Timothy C Fisher, Todd L Graham, Oliver J Buchanan, John S Cambridge, Jonathan K Armstrong, Diane Goldenberg, David A Tanen, James D Ross

**Affiliations:** Critical Innovations LLC, Los Angeles, CA 90260, USA; Department of Emergency Medicine, David Geffen School of Medicine at UCLA, Los Angeles, CA 90095, USA; Department of Emergency Medicine, Harbor-UCLA Medical Center, Torrance, CA 90509, USA; Department of Epidemiology, UCLA—Fielding School of Public Health, Los Angeles, CA 90095, USA; Critical Innovations LLC, Los Angeles, CA 90260, USA; Military & Health Research Foundation, Laurel, MD 20723, USA; Charles T Dotter Department of Interventional Radiology, Oregon Health & Science University, Portland, OR 97239, USA; Critical Innovations LLC, Los Angeles, CA 90260, USA; Critical Innovations LLC, Los Angeles, CA 90260, USA; Critical Innovations LLC, Los Angeles, CA 90260, USA; Critical Innovations LLC, Los Angeles, CA 90260, USA; Department of Emergency Medicine, David Geffen School of Medicine at UCLA, Los Angeles, CA 90095, USA; Department of Emergency Medicine, Harbor-UCLA Medical Center, Torrance, CA 90509, USA; Military & Health Research Foundation, Laurel, MD 20723, USA; Charles T Dotter Department of Interventional Radiology, Oregon Health & Science University, Portland, OR 97239, USA; Center for Regenerative Medicine, Oregon Health & Science University School of Medicine, Portland, OR 97239, USA

## Abstract

**Introduction:**

Noncompressible torso hemorrhage is the leading cause of exsanguination on the battlefield. A self-expanding, intraperitoneal deployed, thermoreversible foam has been developed that can be easily administered by a medic in austere settings to temporarily tamponade noncompressible torso hemorrhage. The purpose of this study was to assess the long-term safety and physical characteristics of using Fast Onset Abdominal Management (FOAM; Critical Innovations LLC) in swine.

**Materials and Methods:**

Yorkshire swine (40-60 kg) were sedated, intubated, and placed on ventilatory support. An external jugular catheter was placed for sampling of blood. Continuous heart rate, temperature, saturation of peripheral oxygen, end-tidal carbon dioxide, and peak airway pressures were monitored for a 4-hour period after intervention (i.e., FOAM agent injection or a sham introducer without agent delivery). The FOAM agent was injected to obtain an intra-abdominal pressure of 60 mmHg for at least 10 minutes. After 4 hours, the animals were removed from ventilatory support and returned to their housing for a period of 7-14 days. Group size analysis was not performed, as this was a descriptive safety study. Blood samples were obtained at baseline and at 1-hour post-intervention and then on days 1, 3, 7, and 14. Euthanasia, necropsy, and harvesting of samples for histologic analysis (from kidneys, terminal ilium, liver, pancreas, stomach, spleen, and lungs) were performed upon expiration. Histologic scoring for evidence of ischemia, necrosis, and abdominal compartment sequela was blinded and reported by semi-quantitative scale (range 0-4; 0 = no change, 1 = minimal, 2 = mild, 3 = moderate, and 4 = marked). Oregon Health & Science University’s Institutional Animal Care and Use Committee, as well as the U.S. Army Animal Care and Use Review Office, approved this protocol before the initiation of experiments (respectively, protocol numbers IP00003591 and MT180006.e002).

**Results:**

Five animals met *a priori* inclusion criteria, and all of these survived to their scheduled endpoints. Two animals received sham injections of the FOAM agent (one euthanized on day 7 and one on day 14), and three animals received FOAM agent injections (one euthanized on day 7 and two on day 14). A transitory increase in creatinine and lactate was detected during the first day in the FOAM injected swine but resolved by day 3. No FOAM agent was observed in the peritoneal cavity upon necropsy at day 7 or 14. Histologic data revealed no clinically relevant differences in any organ system between intervention and control animals upon sacrifice at day 7 or 14.

**Conclusions:**

This study describes the characteristics, survival, and histological analysis of using FOAM in a porcine model. In our study, FOAM reached the desired intra-abdominal pressure endpoint while not significantly altering basic hematologic parameters, except for transient elevations of creatinine and lactate on day 1. Furthermore, there was no clinical or histological relevant evidence of ischemia, necrosis, or intra-abdominal compartment syndrome. These results provide strong support for the safety of the FOAM device and will support the design of further regulatory studies in swine and humans.

## BACKGROUND

Noncompressible torso hemorrhage (NCTH) is the leading cause of exsanguination on the battlefield.^1–3^ In a study of over 15,000 battlefield injuries in Iraq and Afghanistan, 12.7% were torso injuries and 17% of these had need for urgent hemorrhage control.^2^ The military doctrine of forward positioning of surgeons to perform damage control surgery has significantly decreased the time from injury to hemorrhage control and has been cited as one of the reasons for the improved battlefield mortality rates in Iraq and Afghanistan.^4,5^ As an adjunct to damage control surgery, there have also been major advances in the use of endovascular balloon devices for hemorrhage control.^6,7^ However, medics on the battlefield are still limited to applying direct pressure, which is often ineffective. As a result, many injured soldiers die before reaching advanced medical care.^3,8,9^ A new system that is easily accessible to the battlefield medic is needed to temporarily tamponade intra-abdominal bleeding and provide time for casualties to reach higher echelons of care for damage control interventions.^8,9^

**FIGURE 1.**
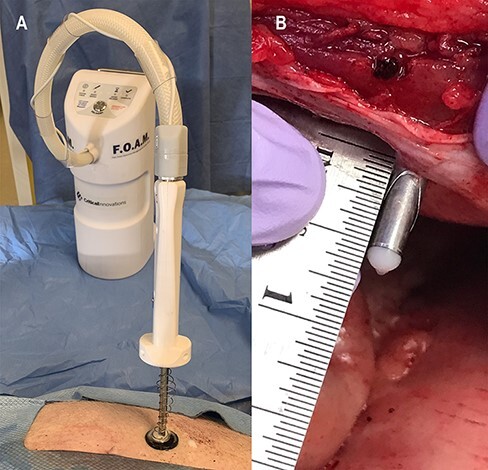
Fast Onset Abdominal Management (FOAM) portable delivery device. The device is designed to control intra-abdominal hemorrhage in the out-of-hospital, prolonged field care, and forward surgical environments (A). The intra-abdominal stopping mechanism auto-senses and auto-stops at the appropriate depth once it passes through the abdominal wall and reaches the peritoneal cavity (B).

A medical device company (Critical Innovations LLC) is developing a new device, Fast Onset Abdominal Management (FOAM), that delivers a self-expanding, intraperitoneal deployed, thermoreversible agent.^10^ FOAM is designed to be transported and administered by medics with minimal training (Fig. 1A). It includes a simplified access mechanism that auto-stops needle penetration once reaching the peritoneal cavity, due to the novel connection of a traditional Veress needle system with a stabilizer flush on the skin that locks to prevent forward movement once sensing access. Thus, the needle is designed to automatically stop at the same penetration depth, regardless of abdominal wall thickness (Fig. 1B).

When deployed, FOAM provides pressure throughout the peritoneal cavity to quickly tamponade NCTH and has been shown to significantly improve mean arterial pressures in a short-term animal efficacy model of surgically induced grade IV liver laceration.^10^ The purpose of this pilot study was to assess the long-term safety and physical characteristics of FOAM deployment in noninjured swine. Specifically, the study explored the ability to clear intraperitoneal deployed agent as well as the effects of increased intra-abdominal pressure generated by the FOAM system on abdominal organs.

## METHODS

Oregon Health & Science University’s Institutional Animal Care and Use Committee, as well as the U.S. Army Animal Care and Use Review Office, approved this protocol before the initiation of experiments (respectively, protocol numbers IP00003591 and MT180006.e002). The care and handling of animals were in accordance with the National Institutes of Health guidelines for ethical animal research. Yorkshire swine (40-60 kg) from a single source vendor (Oak Hill Genetics, Ewing, IL) were sedated with tiletamine and zolazepam (6-8 mg/kg) and 0.005 mg/kg of glycopyrrolate IM. Anesthesia was induced via facemask with 5% isoflurane and 100% oxygen. Animals were then endotracheally intubated and placed on ventilatory support, with an initial tidal volume of 8 mL/kg. Respiratory rate and tidal volume were adjusted to maintain an end-tidal carbon dioxide (EtCO_2_) value between 38 and 42 mmHg and isoflurane maintenance at 1%-3%. Intramuscular buprenorphine was given for additional analgesia.

Access to the external jugular vein was achieved by a percutaneous technique and cannulated for intravenous fluid and blood sampling. After instrumentation, animals were allowed to stabilize for 10 minutes, and baseline blood samples were drawn. Animals were randomly assigned to control sham injections (two animals) or intervention FOAM agent injections (five animals), with planned sacrifice and necropsy on day 7 (for one sham and two FOAM agent injections) and on day 14 (one sham and three FOAM agent injections).

At time 0, the device’s custom, auto-stopping needle introducer was placed into the peritoneal cavity according to the device’s draft Instructions For Use (IFU). For FOAM intervention animals, researchers followed the device’s IFU and utilized its electronic interface to confirm appropriate needle placement and deploy FOAM agent. The device is capable of measuring pressure at its needle tip and was set to automatically deploy FOAM agent to an intra-abdominal pressure of 60 mmHg and then hold that pressure, by dispensing additional agent as needed, over a total time of 10 minutes. After that time, the needle was then removed according to the device IFU, leaving any deployed agent to remain in the peritoneal cavity. For sham animals, the custom, auto-stopping device needle introducer was placed into the abdomen, but no agent was administered and therefore no intra-abdominal pressure was applied.

Continuous heart rate, temperature, EtCO_2_, and saturation of peripheral oxygen were monitored for 4 hours from time 0. Based on previous data showing a strong correlation between airway and intra-abdominal pressure, airway pressures were also monitored as a surrogate for intra-abdominal pressures.^10^ At the end of the 4-hour period, all catheters were removed, and the animals were recovered and returned to their regular housing. Post-intervention blood samples for potassium, pH, amylase, Aspartate transferase (AST), creatinine, and lactate were obtained at 1 hour and then on days 3, 7, and 14. The animals received normal feeding and care until their scheduled sacrifice date (i.e., 7 or 14 days), at which point they were euthanized. Animals were evaluated by lab staff once a day for pain using an *a priori* pain chart based on measures of posture, interaction with surroundings, appetite, activity, and attention to the abdomen on palpation and administered additional analgesia as required. Upon euthanasia, necropsy and collection of samples for histologic analysis (from kidneys, terminal ilium, liver, pancreas, stomach, spleen, and lungs) were performed. A pathologist, who was blinded to the study, was provided with organs from the necropsies and used a predetermined semi-quantitative scale for the determination of evidence of ischemia, necrosis, and abdominal compartment sequela (range 0-4; 0 = no change, 1 = minimal, 2 = mild, 3 = moderate, and 4 = marked).

## RESULTS

An *a priori* power analysis to determine group size was not performed, as this was a descriptive safety study. Seven male swine started the study with an average weight of 48.6 ± 5.9 kg. According to inclusion/exclusion criteria established *a priori*, two animals were sacrificed for necropsy when the FOAM device failed to reach the predetermined intra-abdominal pressure. Of the remaining five animals, all survived to their scheduled endpoint.

**FIGURE 2.**
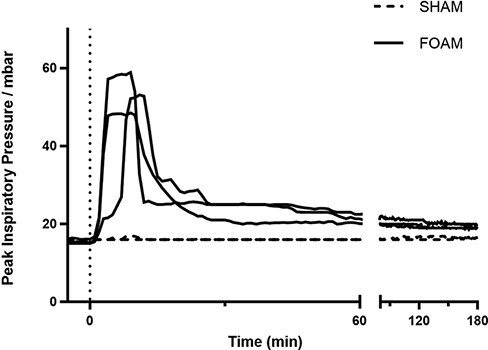
Airway pressures measured over time while swine were mechanically ventilated. Airway pressure from time 0 to 4 hours was elevated in animals receiving FOAM as compared to sham controls. mbar = millibar. min = minutes.

**FIGURE 3.**
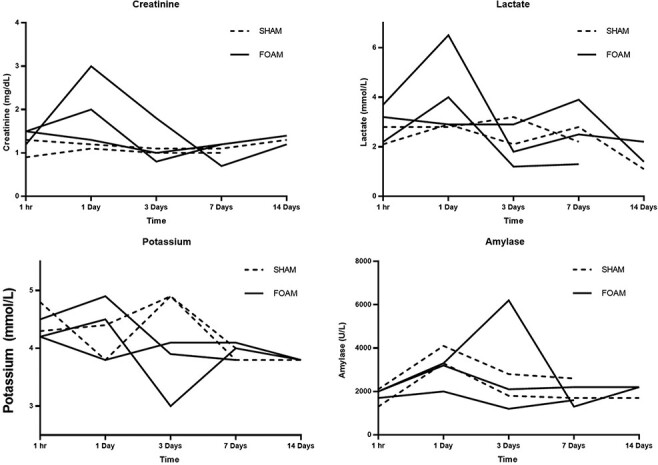
Measurement of creatinine, lactate, potassium, and amylase over time. A transient increase in creatinine and lactate was detected during the first day in the FOAM injected swine but resolved by day 3. mg/dL = milligrams per deciliter. mmol/L = millimoles per liter. U/L = units per liter.

Two animals received sham injections of FOAM agent (one euthanized on day 7 and one on day 14), and three animals received FOAM agent injections (one euthanized on day 7 and two on day 14). There were no clinical differences observed between animals at baseline in terms of heart rate, amount of isoflurane used to maintain sedation, or ventilator settings adjusted to maintain EtCO_2_. Airway pressure, which was used as a surrogate for abdominal pressure monitoring, was found to be elevated in animals receiving FOAM as compared to sham controls, especially within the first 60 minutes, with the intervention pressures trending closer to controls by around 120-180 minutes (Fig. 2). A transient increase in creatinine and lactate was detected during the first day in the FOAM injected swine but resolved by day 3 (Fig. 3).

After recovery, the two animals that received sham injections did not require additional analgesia. Two FOAM animals required one dose of analgesia and one animal required two doses of analgesia. At necropsy, no FOAM agent was observed in the peritoneal cavity at day 7 or 14 for any animal. Histologic data revealed no observable clinical differences in any organ system between intervention and control animals upon sacrifice at days 7 or 14 (Table I).

**TABLE I.** FOAM versus Sham Injected Animals

		Sham at 7 days	Sham at 14 days	FOAM at 7 days	FOAM at 14 days	FOAM at 14 days
Lung	Hemorrhage	3	3	2	3	3
	Edema	1	1	0	2	1
	Inflammation	2	3	2	2	1
	Vasculitis	2	2	2	2	2
	Necrosis	0	0	0	0	0
Spleen	Hemorrhage	0	0	0	0	0
	Edema	0	0	0	0	0
	Inflammation	0	0	0	0	0
	Vasculitis	0	0	0	0	0
	Necrosis	0	0	0	0	0
Pancreas	Hemorrhage	0	0	0	0	0
	Edema	0	0	0	0	0
	Inflammation	0	0	0	0	0
	Vasculitis	0	0	0	0	0
	Necrosis	0	1	0	0	0
Liver	Hemorrhage	2	3	2	2	2
	Edema	3	3	2	2	2
	Inflammation	1	2	1	2	2
	Vasculitis	0	0	0	0	0
	Necrosis	2	3	2	2	2
Kidney	Hemorrhage	2	2	2	1	1
	Edema	0	0	0	0	0
	Inflammation	2	0	1	1	1
	Vasculitis	0	0	0	0	0
	Necrosis	3	2	1	2	2

Results of the histologic analysis of harvested organs at necropsy for FOAM versus sham injected animals using a scale to measure effects of ischemia (range 0-4; 0 = no change, 1 = minimal, 2= mild, 3 = moderate, and 4 = marked).

## DISCUSSION

A simple and easily transportable system to tamponade NCTH is needed for both the medic on the battlefield and the civilian emergency provider with any significant delay to surgical intervention.^3,9^ A prior study has demonstrated that there is an approximately 1% increase in mortality for each 3-minute delay to surgery in such patients.^5^ Although there have been marked improvements in the use of damage control surgery, resuscitation, and the development of endovascular devices, many patients still die of exsanguination during transport from the scene of the injury or during delays to definitive surgical intervention.^1,3,6^ These wounds are, by definition, noncompressible in the context of current technology, and FOAM may represent a unique method to temporize such severely injured patients.

FOAM was developed purposefully in response to acknowledged safety concerns from previous devices under development for NCTH management. A previous effort (ResQFoam) formed a hard solid once deployed that had to be physically removed by a surgeon afterward, while producing focal areas of bowel ischemia in a swine model.^11^ In contrast, the FOAM device utilizes a thermoreversible, reverse-phase-changing polymer mixture with an inert expanding gas that, when deployed, forms a softer gel to exert tamponade on bleeding organs. Previous studies of these polymers show that they are cleared by body through the kidney over a 48- to 72-hour time frame, when administered intravenously.^12,13^ This is the first study to demonstrate that they are also successfully removed by the body from the peritoneal cavity.

Similar to previous traumatic hemorrhage research, this study also utilized swine, due to multiple similarities with human physiology.^14,15^ Although providing likely a higher intra-abdominal pressure than past efforts, there were no signs of significant ischemic bowel injury or intra-abdominal compartment syndrome in this long-term study. The FOAM device transiently elevates intra-abdominal pressures through the automated, pulse deployment of agent in connection with pressure sensing capability from the needle’s tip and was set to a goal pressure of 60 mmHg. Deployment occurred for a period of 10 minutes, after which the needle was removed, while the deployed agent continued to exert pressure internally. Previous studies have demonstrated that the intra-abdominal pressure tracks closely with peak inspiratory pressure when using this technology in this model.^10^ Thus, Figure 2 shows that the FOAM device initially elevated swine intra-abdominal pressures to around 50-60 mmHg. The pressure subsequently remained elevated for the next 60-120 minutes, with slow decayed back toward normal afterward.

In our limited sample of swine, this deployment and elevated intra-abdominal pressure led to a transient increase in lactate and creatinine. However, importantly we did not find any histologic evidence of compartment syndrome, pressure necrosis, or tissue-threatening ischemia. This evidence strongly supports device design that, similar to the use of tourniquets for extremity hemorrhage, seeks to apply significant temporary pressure to induce hemorrhage tamponade, while removing that pressure before tissue compression leads to significant ischemia and long-term sequelae.

There are significant limitations to this pilot study and to interpreting animal data as it relates to human pathophysiology. To minimize these, we chose a moderately sized swine model (40-60 kg) based on previous investigations of traumatic abdominal injuries.^14,15^ To address specific limitations, although we did not include an intra-abdominal pressure monitor, we did follow airway pressures that have been shown to correlate with abdominal pressures.^16,17^ Although not validated, the histologic scale used for this study utilized a standardized numerical rating scale for ischemia.

## CONCLUSIONS

This study describes the characteristics, survival, and histological analysis of using FOAM in a porcine model. In our study, FOAM reached the desired intra-abdominal pressure endpoint while not significantly altering basic hematologic parameters, except for transient elevations of creatinine and lactate on day 1. Furthermore, there was no clinical or histological relevant evidence of ischemia, necrosis, or intra-abdominal compartment syndrome. These results provide strong support for the safety of the FOAM device and will support the design of further regulatory studies in swine and humans.
